# Within- and between-individual associations between sleep and cognition in older community-dwelling individuals

**DOI:** 10.3389/fragi.2025.1650312

**Published:** 2026-01-22

**Authors:** Ciro della Monica, Kiran K. G. Ravindran, Giuseppe Atzori, William Trender, Adam Hampshire, Simon S. Skene, Hana Hassanin, Victoria Revell, Derk-Jan Dijk

**Affiliations:** 1 Surrey Sleep Research Centre, School of Biosciences, Faculty of Health and Medical Sciences, University of Surrey, Guildford, United Kingdom; 2 UK Dementia Research Institute, Care Research and Technology Centre at Imperial College, London and the University of Surrey, Guildford, United Kingdom; 3 Department of Brain Sciences, UK Dementia Research Institute Care Research and Technology Centre, Imperial College London, London, United Kingdom; 4 Department of Neuroimaging, King’s College London, London, United Kingdom; 5 Surrey Clinical Trials Unit, School of Biosciences, Faculty of Health and Medical Sciences, University of Surrey, Guildford, United Kingdom; 6 Surrey Clinical Research Facility, University of Surrey, Guildford, United Kingdom; 7 NIHR Royal Surrey CRF, Guildford, United Kingdom

**Keywords:** aging, cognition, community-dwelling, individual variation, sleep

## Abstract

**Introduction:**

Cross-sectional and interventional studies have demonstrated that sleep has a significant impact on waking brain function, including alertness and cognitive performance. Few studies have assessed whether spontaneous night-to-night variation in sleep is associated with variation in brain function within an individual. How this compares to inter-individual variation in sleep and cognition and their associations also remains largely unknown. These questions are of particular interest in the context of aging because both sleep and cognitive abilities are altered.

**Methods:**

Furthermore, older people have been reported to be less sensitive to sleep loss. Here, we investigated the relationship between sleep and cognition by quantifying associations between intra-individual variation in sleep and cognition, along with associations between inter-individual variation in sleep and cognition, in 35 cognitively intact older adults (70.8 ± 4.9 years; mean ± SD; 14 female individuals) living in the community. Subjective and actigraphic sleep measures and daily digital assessments of cognition (9 cognitive tests; 19 variables) were obtained over a 2-week period. The cognitive test battery probed a wide range of cognitive functions, including reaction time, working memory, attention, and problem-solving. Principal component analysis (PCA) identified four principal sleep components, namely, sleep duration, sleep efficiency, subjective sleep quality, and nap effect. Mixed model analyses were conducted with mean and deviation-from-the-mean cognitive variables to quantify how inter- and intra-individual variations in sleep were associated with inter- and intra-individual variations in cognition.

**Results:**

Longer sleep duration was associated with faster reaction times in both the inter- and intra-individual analyses and with reduced errors in the inter-individual analyses. Higher sleep efficiency was associated with faster reaction times in both the intra- and inter-individual analyses. In contrast, aspects of cognition relating to learning, visual memory, verbal reasoning, and verbal fluency were not associated with sleep.

**Discussion:**

These data show that, in older people, some aspects of waking function are sensitive to night-to-night variation in sleep duration and efficiency, implying that interventions targeting these aspects of sleep may be beneficial for waking function in aging.

## Introduction

Sleep has a significant impact on various aspects of waking function, including alertness, mood, and cognition. The relationship between sleep and waking brain function is, however, complex and multifaceted and has been studied in different study designs. The methods used to assess sleep and cognition vary widely across these studies, ranging from self-reported sleep and cognitive function to polysomnography with quantitative electroencephalogram (EEG) analyses and cognitive function test batteries that probe a range of cognitive domains.

Numerous studies have reported the effects of acute total sleep deprivation on waking function (Van Dongen et al., 2003; Rusterholz et al., 2017), whereas fewer studies have used repeated partial sleep deprivation (Lo et al., 2012; Philip et al., 2012; Maric et al., 2017; Banks et al., 2024) or designs in which specific sleep stages, for example, slow-wave sleep (SWS) or rapid eye movement (REM) sleep, were manipulated (Groeger et al., 2014; Navarrete et al., 2024). These interventional studies have established the contribution of sleep to daytime alertness, sustained attention, and positive affect, while also providing evidence for a role of sleep in memory consolidation (Hornung et al., 2007; Koa and Lo, 2021; Groeger et al., 2022). Non-interventional studies, such as cross-sectional studies, have quantified associations between variation in sleep and variation in waking function across participants (della Monica et al., 2018). As an alternative to these interventional and cross-sectional designs, within-participant study designs, in which sleep and cognition are assessed daily for days or weeks, can be used to assess the impact of night-to-night variation in sleep on day-to-day waking function within individuals.

Interventional, cross-sectional, longitudinal, and short-term within-participant designs are all valuable in understanding the role of sleep in brain function across the lifespan or in specific age groups such as older people. However, for a given individual, understanding the associations between their night-to-night variations in sleep and daytime function is most relevant to them. This is because spontaneous variation in aspects of sleep is, by definition, within an ecologically relevant range. Understanding how within-participant variations in sleep (driven by endogenous or exogenous factors, e.g., light exposure and behavioral factors) are associated with waking function may identify targets for personalized sleep interventions (Skeldon et al., 2023). Within these designs, variability itself (such as variability in sleep timing) can be considered an independent variable. Two recent studies have explored the impact of night-to-night variability in sleep metrics on next-day cognitive performance in both older adults and cognitively impaired older adults to assess both inter- and intra-individual variations (Balouch et al., 2022; Schwarz et al., 2025). In addition, variability in sleep has also been demonstrated in relation to cognition and Alzheimers disease (AD) pathology (Fenton et al., 2023), and odds of developing cognitive impairment (Diem et al., 2016) have also been demonstrated. Moreover, a relationship between sleep structure, AD pathology, and sleep-dependent memory consolidation has been demonstrated (Mander et al., 2022). Longitudinal study designs allow for comparison of the strength of inter-individual associations between sleep and cognition (i.e., trait-like associations) with the magnitude of intra-individual associations.

How sleep is associated with cognitive function is of particular interest in aging because both sleep and cognition show marked changes, and inter-individual variation in sleep and cognition increases with age (Djonlagic et al., 2021; Qin et al., 2023). Furthermore, several studies have suggested that older people may become less affected by poor sleep or sleep loss induced by total or repeated partial sleep deprivation (Adam et al., 2006; Dijk et al., 2010; Sagaspe et al., 2012; Zitting et al., 2018).

Age-related changes in various aspects of sleep and in a range of cognitive domains have been well established in cross-sectional studies, with age-related reductions in slow-wave sleep and processing speed being prominent examples (Mander et al., 2017). Cross-sectional studies have also addressed how age-related changes in sleep are associated with those in cognition and whether these associations persist when age itself is controlled for as a general modifying or confounding factor. In one such cross-sectional polysomnographic study on 206 healthy adults aged 20–84 years, it was confirmed that SWS changes the most with aging (della Monica et al., 2018). In this study, cognition was assessed across cognitive domains, such as sustained attention, response time, motor control, working memory, and executive function, and the largest age-related effects were observed for aspects of ‘speed.’ After controlling for age and sex, it was found that inter-participant variation in SWS contributed to individual differences in processing speed. However, variation in REM sleep and, in particular, the number of awakenings, both of which did not show marked age-related changes, explained inter-participant variation in accuracy and aspects of executive function (della Monica et al., 2018). Similarly, a much larger study by Djonlagic et al. (2021), which applied standard polysomnographic and advanced quantitative EEG measures but was limited to older participants, demonstrated that, for macro-architectural aspects of sleep, after controlling for age and sex, increased REM sleep duration and sleep efficiency were positively associated with cognition as assessed by the Digit Symbol Substitution and Trails Making B test, with little evidence for a contribution of SWS. A meta-analysis of how, in older people, polysomnographically assessed sleep relates to cognition identified REM sleep and sleep continuity as positive predictors (Qin et al., 2023), whereas SWS was not a significant contributor. Studies in which sleep assessment was based on actigraphy have also highlighted the role of sleep continuity (Owusu et al., 2023).

Few studies have investigated how night-to-night variation in sleep is associated with day-to-day variation in aspects of waking function in aging. Master et al. (2023) demonstrated associations between night-to-night variation in sleep duration and mood in adolescents. Gamaldo et al. (2010) reported associations between both intra- and inter-participant variation in sleep duration and a range of cognitive functions in older African Americans; however, sleep duration was based solely on self-report. In another recent study on older people with and without dementia (Balouch et al., 2022), it was found that night-to-night variation in sleep, as assessed through actigraphy and sleep diary and represented by its principal components, i.e., duration, quality, continuity, and latency, was associated with day-to-day variation in alertness, daily memory errors, serial subtraction, and behavioral problems. Night-to-night variations in sleep duration and, in particular, sleep continuity measures were the most powerful predictors of intra-participant variation in daytime performance (Balouch et al., 2022). In that study, intra- and inter-participant associations were not compared, and the relevance of sleep variability itself for daytime function was not assessed.

Such longitudinal studies are often limited in the number of cognitive tests that are used. Digital platforms of cognitive testing offer a scalable solution that can be used longitudinally by individuals in their own homes without the need to interact with researchers. Such approaches have been used successfully, repeatedly, over months or years in a variety of clinical populations, including people living with traumatic brain injury (Brooker et al., 2020; Hampshire et al., 2022; Peers et al., 2022; Lennon et al., 2023; Stewart et al., 2023). To date, digital cognitive testing has not been administered on a daily basis in conjunction with objective assessment of sleep in aging.

In this study, we used daily, self-administered, digitally assessed measures of cognitive function at home over a 2-week period while concurrently assessing sleep by self-report and objectively using actigraphy. The test battery assessed a wide range of cognitive functions, including reaction time, working memory, attention, problem-solving, and verbal fluency. One aim of the study was to assess the feasibility and acceptability of daily digital assessments of cognitive performance in conjunction with daily assessments of sleep. In addition, we aimed to investigate whether (and which) parameters of objective and subjective sleep are associated with inter-participant variation in parameters of cognitive performance in older adults and whether night-to-night variation in objective and subjective sleep measured at home predicts day-to-day variation in digitally assessed cognitive performance, i.e., intra-participant variation and association.

## Methods

### Participants and screening

The inclusion/exclusion criteria for participation in this protocol were designed to ensure a representative heterogeneous population for this age range. Following an initial telephone screening interview, participants attended an in-person screening visit to determine their eligibility, including measurement of height, weight, and vital signs; self-reported medical history; symptom-directed physical examination; and completion of the Epworth Sleepiness Scale (ESS) (Johns, 1991), Pittsburgh Sleep Quality Index (PSQI) (Buysse et al., 1989), Activities of Daily Living Questionnaire (ADL) (Lawton and Brody, 1969), and International Consultation on Incontinence Questionnaire—Urinary Incontinence (ICIQ-UI) (Avery et al., 2004). Participants were deemed eligible for the study if they met defined inclusion/exclusion criteria, including self-declared stable and controlled mental and physical health conditions, the ability to perform daily living activities independently, consumption of <29 units of alcohol per week, and being current non-smokers.

### Study protocol

The study received a favorable opinion from the University of Surrey Ethics Committee (UEC 2019 065 FHMS, 02 August 2019) and was conducted in accordance with the Declaration of Helsinki and guided by the principles of Good Clinical Practice. Written informed consent was obtained from participants before any procedures were performed, and participants were compensated for their time and inconvenience.

The study protocol has been described extensively elsewhere (Ravindran K. et al., 2023; Ravindran K. K. G. et al., 2023; della Monica et al., 2024). In brief, participants attended the Surrey Sleep Research Centre (SSRC) and were provided with a range of devices to use at home for 7–14 days for sleep, circadian, and environmental monitoring. Relevant to this analysis, participants were requested to continually wear an Actiwatch Spectrum (AWS, Philips Respironics, United Kingdom) to monitor rest–activity patterns and environmental light exposure. Participants could only remove the device if it was going to get wet, for example, during showering, swimming, or washing up. In addition, participants were requested to complete the paper Consensus Sleep Diary-M on a daily basis, which includes questions related to sleep timing, quality, and duration; daytime naps; and alcohol and caffeine consumption (Carney et al., 2012). Each morning, approximately 1 hour after waking, participants were requested to complete their daily cognitive testing on an electronic tablet using the bespoke Cognitron testing platform (Hampshire et al., 2021). The daily battery took approximately 30 min to complete and included the following nine tests: 2D manipulations, choice reaction time, simple reaction time, digit span, learning curves, paired associate learning, self-ordered search, verbal reasoning, and word definitions, which probed specific domains. Following this at-home monitoring period, participants attended the Research Centre for an overnight stay, which included a 10-h period in bed with full polysomnography (PSG) recording [in accordance with the Academy of Sleep Medicine (AASM) guidelines], from which we assessed sleep apnea (apnea–hypopnea index, AHI).

### Data analysis

#### Sleep diary

The diary (Carney et al., 2012) was completed on paper, and the recorded information was converted into electronic format and reviewed for completeness and integrity. Nonsensical data were eliminated from further analysis. Sleep estimates were taken directly from the answers provided by participants (e.g., time taken to fall asleep, number of awakenings) or were derived from the answers: time in bed = time out of bed − time get to bed; attempted sleep period = final awakening − time got to sleep; sleep period = final awakening − time got to sleep − time taken to fall asleep; and sleep efficiency = (final awakening − time got to sleep − time taken to fall asleep − how long awake)/final awakening − time got to sleep)*100.

#### Actiwatch

Activity data were collected at 1-min epochs and analyzed using the proprietary algorithm in Actiware 6.0.7 software. Each epoch was assigned as either sleep or wake using the medium sensitivity threshold (40 activity counts), and sleep summary estimates were derived. In accordance with the AASM guidelines, the analysis interval for the major nocturnal sleep episode for each night was set using the clock times for attempting sleep and final awakening as recorded in the sleep diary (Smith et al., 2018).

The sleep estimates derived from the AWS and sleep diary are shown in Table 1.

**TABLE 1:** Sleep estimates derived from the sleep diary and Actiwatch.

Type of measure	Subjective sleep—sleep diary		Objective sleep—Actiwatch	
	Variable label	Variable description	Variable label	Variable description
Common measures	sTST	Total sleep time (min)	awsTST	Total sleep time (min)
	sSE	Sleep efficiency (%)	awsSE	Sleep efficiency (%)
	sWASO	Wake after sleep onset (min)	awsWASO	Wake after sleep onset (min)
	sSOL	Sleep onset latency (min)	awsSOL	Sleep onset latency (min)
	sNAW	Number of awakenings (number)	awsN_WAKE_BOUTS	Number of awakenings (number)
	sATTEMPTED_SLEEP	Attempted sleep period (min)	awsSLEEP_PERIOD	Attempted sleep period (min)
Measures only subjective or only objective	sTIB	Time in bed (min)	awsWAKE_PERC	Wake %
	sSLEEP_PERIOD	Sleep period (min)	awsAVG_WAKE_BOUTS	Average duration of wake bouts (min)
	sQoS	Quality of sleep (number, Likert scale)	awsSLEEP_PERC	Sleep %
	sRuA	Refreshed upon awakening (number, Likert scale)	awsAVG_SLEEP_BOUTS	Average duration of sleep bouts (min)
	sTOT_NAP_TIME	Total nap time (min)	awsN_SLEEP_BOUTS	Number of sleep bouts (number)
	sN_NAP	Number of naps (number)	​	​
	sSLEEP_AFTER_ FINAL_AWAKE	How long did you try to sleep after your final awakening (min)?	​	​
	sEARLIER_WAKEUP	Did you wake up earlier than planned (min)?	​	​
	sHOW_MUCH_EARLIER	If so, how much earlier (min)?	​	​
	sN_ALCOH_DRINKS	Number of alcoholic drinks consumed (number)	​	​
	sN_CAFF_DRINKS	Number of caffeinated drinks consumed (number)	​	​
	sSLEEP_DRUG	Taken any over-the-counter or prescribed medication (Y/N)	​	​
	sGOTOSLEEP	Clock time of time asleep (min after midnight)	​	​
	sFINALAWAKE	Clock time of final awakening (min after midnight)	​	​

#### Cognitive test battery

The data were available in. csv format for each test separately for each day and each participant. These files were subsequently processed to extract variables related to speed and accuracy for each test (Table 2).

**TABLE 2:** Cognitive tests used with the domain that they probe and the variables generated.

Cognitive test name	Domain	Variable label	Variable description
Choice reaction time task	Speed–accuracy trade off	CHOICE_RT_MEAN_RT_for_CORRECT	Mean reaction time for correct trials (msec)
		CHOICE_RT_ERRORS	Number of errors (number)
		CHOICE_RT_STD	Standard deviation reaction time (msec)
Digit span task	Working memory and attention	DIGIT_SPAN_N_MAX_ACHIEVED	Maximum span achieved (number)
		DIGIT_SPAN_MEDIAN_RT	Median reaction time (msec)
Learning curve task	Learning and memory	LEARNING_CURVES_MEDIAN_RT	Median reaction time (msec)
		LEARNING_CURVES_TOT_CORRECT	Total correct number (number)
Paired associate learning task	Visual memory and new learning	PAIRED_ASSOC_LEARN_N_MAX_ACHIEVED	Maximum span achieved (number)
Self-ordered search task	Working memory and frontal	SELF_ORDERED_SEARCH_N_MAX_ACHIEVED	Maximum level achieved (number)
		SELF_ORDERED_SEARCH_ERRORS	Number of errors (number)
Simple reaction time task	Reaction time	SIMPLE_RT_MEAN	Mean reaction time (msec)
		SIMPLE_RT_ERRORS	Number of errors (number)
		SIMPLE_RT_STD	Standard deviation reaction time (msec)
Verbal reasoning	Problem solving and verbal reasoning	VERBAL_REAS_TOT_CORRECT	Total correct number (number)
		VERBAL_REAS_MEDIAN_RT_for_CORRECT	Median reaction time for correct trials (msec)
Word definitions	Memory and verbal fluency	WORD_DEFIN_TOT_CORRECT	Total correct number (number)
		WORD_DEFIN_MEDIAN_RT	Median reaction time (msec)
2D manipulation	Reasoning and abstract thinking	2D_MANIPUL_MEDIAN_RT_for_CORRECT	Median reaction time for correct trials (msec)
		2D_MANIPUL_TOT_CORRECT	Total correct number (number)

### Statistical analysis

Intraclass correlation coefficient (ICC) analysis was performed in R (v. 4.2.2, R Core Team 2022, Psych library, ICC function with two-way random effect) to determine the intra- vs. inter-individual variation using the dependent variable (sleep or cognition) as “response” and participant ID as “subject.”

Principal component analysis (PCA) was then conducted using SAS (v9.4, SAS Institute Inc.) Proc Factor, using the principal method followed by Varimax rotation. The PCA only utilized an imputed dataset for subjective and objective sleep variables. Missing data for each sleep variable (3.6% ± 2.5%, mean ± SD) were assumed to be missing at random due to occasional device data loss or diary omissions that did not depend on the values of the missing variables themselves. Due to the distribution of missing data and the sample size, the approach taken was to impute missing values using the mean value of each variable across participants.

A mixed-model analysis was conducted in SAS using PROC MIXED, in which separate mixed models were fit for each cognitive variable, with the cognitive measure as the dependent variable and sex, age, body mass index (BMI), AHI, time-in study (to account for learning effects), and sleep component as linear fixed effects. This analysis used the non-imputed dataset as the SAS PROC MIXED function can handle missing data. Models included random intercepts for participants, accommodating the correlation between repeated measurements on an individual. Random slopes were not estimated because the number of repeated observations per participant (∼14 days) was insufficient to reliably estimate participant-specific slopes. Sleep components were separated into inter-person (i.e., the individual’s average sleep values across the study period) and intra-person levels (i.e., the deviation of the sleep values from the individual’s average). Both inter- and intra-person levels were simultaneously included in the model (Yap et al., 2024). Individual differences in cognitive ability were modeled as a random effect to account for repeated measures on an individual.

## Results

### Study population

Thirty-five older adults (14 female individuals), aged 65–83 years (70.8 ± 4.9 years; mean ± SD), participated in the study. Participants had a PSQI score of 4.1 ± 2.1, an ESS score of 3.6 ± 2.5, an ICIQ score of 1.0 ± 1.7, an ADL score of 7.9 ± 0.2, a BMI of 26.7 ± 4.7 kg/m^2^, and a Mini-Mental State Examination (MMSE) score of 28.7 ± 1.4 (mean ± SD).

### Data completeness

Of 399 planned days/nights of recording at home, the following data were available for analysis: 1) AWS: 378 days (95% data availability); 2) sleep diary: 395 days (99% data availability); and 3) Cognitron data: 379 days (95% data availability). This high level of data completeness also demonstrates the acceptability of the methodology.

### Longitudinal assessment of sleep and cognition at home

**FIGURE 1:**
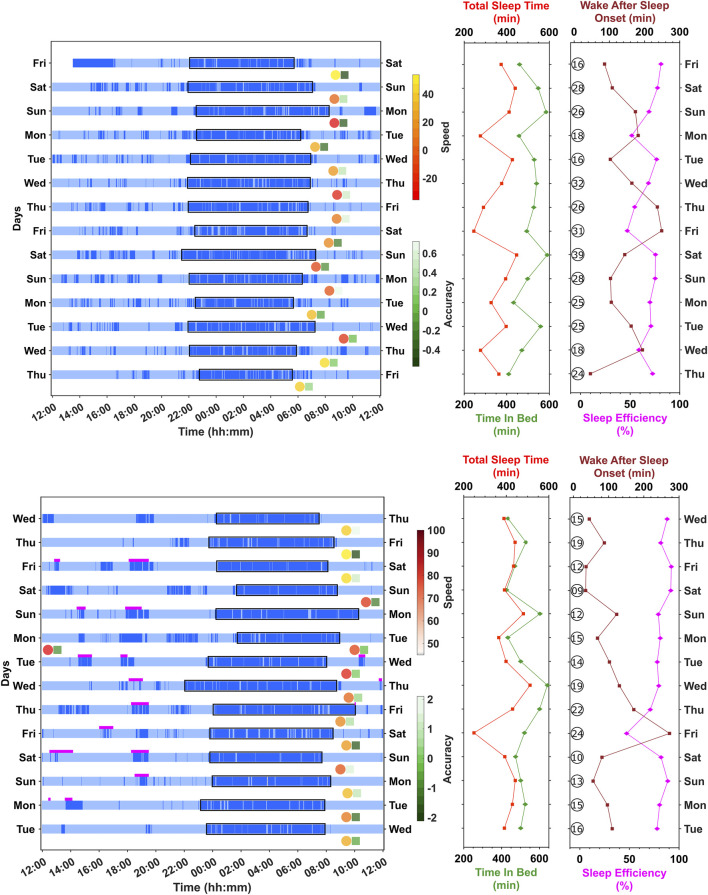
Raster plot of rest/activity (Actiwatch) for two different participants. Top panel—simple reaction time (speed and accuracy); bottom panel—learning curves (speed and accuracy). Dark blue bars indicate periods of rest, and light blue bars indicate periods of activity. The timing of the tasks is indicated by the position of the circle (speed) and square (accuracy) symbols. The spectrum indicates the accuracy or speed relative to the centered mean. For speed, positive values indicate slower reaction time and, therefore, worse performance. For accuracy, in the simple reaction time task, positive values indicate increased errors, whereas with learning curves, positive values indicate a higher number of correct answers. Magenta bars indicate naps reported in the sleep diary (bottom panel only). The right-hand side panels provide sleep metrics per night: time in bed (minutes), total sleep time (minutes), wake after sleep onset (minutes), sleep efficiency (%), and the number of awakenings (numbers in circles).

The raster plots in Figure 1 provide examples of rest/activity, cognitive performance, and sleep parameters over 14 days at home for two participants using the Actiwatch and the Cognitron platform. In the top panel, night-to-night variations in sleep timings and durations are evident, including an increased time in bed on Saturday nights. In addition, reaction times vary from day-to-day and are fastest with increased sleep duration, whereas accuracy variation is not necessarily associated with sleep duration. In the participant shown in the bottom panel, daytime naps are observed, and there is more night-to-night variation in sleep duration. For this individual, day-to-day variations in both speed and accuracy are not obviously associated with sleep duration.

## Descriptive statistics for sleep and cognitive variables

### Intra- and inter-subject variation

**FIGURE 2:**
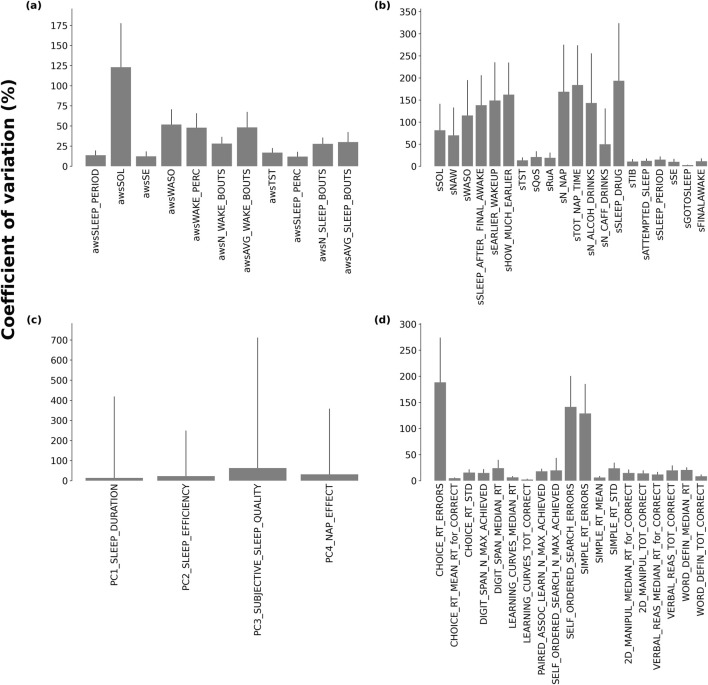
Intra-individual variability for **(a)** objective sleep measures, **(b)** subjective sleep measures, **(c)** sleep principal components, and **(d)** cognitive performance measures. Variability is expressed as coefficient of variation (CV) computed as the average of CVs per participant. Error bars indicate standard deviations.

Examples of inter- and intra-individual variation in objective and subjective sleep measures, along with cognitive performance measures, are shown in Supplementary Figure S1. Average actigraphically determined sleep efficiency varied greatly between participants, ranging from 37.32% to 95.05%, but also varied considerably within participants (coefficient of variation = 19.89%; range = 57.73). Visual inspection of objective and subjective sleep efficiency did not reveal any obvious pattern across the 14 days. In contrast, the number correct on the verbal reasoning task showed improvement over the 14 days. Intra-individual variability is depicted in Figure 2, where the coefficients of variation are shown for the individual sleep and cognitive variables.

ICCs were computed for all the sleep and cognitive variables (Tables 3, 4). Values closer to 1 indicate that values for a participant are consistent, and inter-individual variation is higher than intra-individual variation; values closer to 0 indicate that the values for a participant are highly variable, and there is a higher contribution of intra-individual variation. For the sleep variables, the ICCs ranged from 0.11 to 0.86, with the lower values related to subjective assessments of sleep onset latency (sSOL), wake after sleep onset (sWASO), and sleep efficiency (sSE). Higher ICCs were observed for objective measures, including sleep efficiency (awsSE), number of awakenings (awsN_WAKE_BOUTS), and WASO (awsWASO). For the cognitive variables, the ICCs ranged from 0.19 to 0.92, with the highest values (>0.75) observed for reaction time variables [e.g., choice RT mean (correct trials), simple RT mean, and learning curve RT median) and the lowest predominantly for accuracy (e.g. learning curve total correct, self-ordered search errors, and paired associate maximum score).

**TABLE 3:** Intra-class correlations for objective and subjective sleep variables.

Variable (unit of measurement)	Mean	Standard deviation	ICC (95% CI)
sSE (%)	88.86	11.2	0.11 (0.05–0.21)
sWASO (min)	28	40.45	0.12 (0.06–0.23)
sSOL (min)	25.07	40.45	0.14 (0.07–0.26)
sEARLIER_WAKEUP (min)	0.35	0.48	0.25 (0.16–0.39)
sTOT_NAP_TIME (min)	9.59	25.46	0.27 (0.18–0.42)
sSLEEP_AFTER_ FINAL_AWAKE (min)	27.02	52.33	0.33 (0.22–0.47)
sHOW_MUCH_EARLIER (min)	20.27	40.18	0.34 (0.23–0.49)
awsSOL (min)	11.93	21.28	0.35 (0.24–0.5)
sN_ALCOH_DRINKS (number)	0.55	0.85	0.36 (0.25–0.51)
sQoS (number)	3.3	0.95	0.39 (0.28–0.54)
sN_NAP (number)	0.31	0.57	0.41 (0.29–0.56)
awsAVG_WAKE_BOUTS (min)	4.48	3.2	0.48 (0.36–0.63)
sNAW (number)	1.95	1.58	0.48 (0.36–0.63)
sSLEEP_PERIOD (min)	417.11	92.67	0.49 (0.37–0.63)
sRuA (number)	3.18	0.92	0.54 (0.42–0.68)
sTST (min)	403.77	82.29	0.54 (0.42–0.68)
awsTST (min)	330.39	82.41	0.55 (0.43–0.69)
sSLEEP_DRUG (Y/N)	0.05	0.22	0.56 (0.44–0.7)
sTIB (min)	497.67	83.08	0.57 (0.45–0.7)
awsAVG_SLEEP_BOUTS (min)	20.64	12.19	0.57 (0.45–0.71)
awsSLEEP_PERIOD (min)	419.56	91.75	0.59 (0.47–0.72)
sATTEMPTED_SLEEP (min)	442.32	88.31	0.6 (0.48–0.73)
awsN_SLEEP_BOUTS (number)	20	9.09	0.61 (0.49–0.74)
awsSLEEP_PERC (%)	79.91	14.85	0.62 (0.5–0.75)
awsWAKE_PERC (%)	20.09	14.85	0.62 (0.5–0.75)
awsN_WAKE_BOUTS	19.42	9.02	0.63 (0.51–0.75)
sFINALAWAKE (min after midnight)	408.65	78.58	0.63 (0.51–0.75)
awsWASO (min)	88.93	72.74	0.63 (0.52–0.76)
awsSE (%)	75.79	15.07	0.65 (0.54–0.77)
sGOTOSLEEP (min after midnight)	1406.26	62.88	0.73 (0.63–0.83)
sN_CAFF_DRINKS (number)	3.03	1.99	0.86 (0.8–0.92)

**TABLE 4:** Intra-class correlations for cognitive variables.

Variable	Mean	Standard deviation	ICC (95% CI)
DIGIT_SPAN_MEDIAN_RT (msec)	7821.44	2799.14	0.19 (0.11–0.31)
LEARNING_CURVES_TOT_CORRECT (number)	117.7	3.37	0.22 (0.14–0.36)
SELF_ORDERED_SEARCH_ERRORS (number)	1.52	2.3	0.24 (0.14–0.38)
PAIRED_ASSOC_LEARN_N_MAX_ACHIEVED (number)	3.9	0.83	0.24 (0.15–0.38)
PAIRED_ASSOC_LEARN_N_MAX_ACHIEVED (number)	1.3	2	0.28 (0.18–0.43)
CHOICE_RT_ERRORS (number)	0.57	1.34	0.32 (0.21–0.47)
CHOICE_RT_STD (msec)	82.94	18.97	0.49 (0.37–0.64)
SIMPLE_RT_STD (msec)	72.25	25.96	0.54 (0.42–0.69)
DIGIT_SPAN_N_MAX_ACHIEVED (number)	7.26	1.79	0.56 (0.44–0.7)
WORD_DEFIN_TOT_CORRECT (number)	17.75	2.31	0.56 (0.44–0.7)
VERBAL_REAS_TOT_CORRECT (number)	31.06	10.26	0.67 (0.56–0.79)
SELF_ORDERED_SEARCH_N_MAX_ACHIEVED (number)	7.25	2.04	0.68 (0.57–0.79)
2D_MANIPUL_TOT_CORRECT (number)	24.94	6.88	0.73 (0.63–0.83)
WORD_DEFIN_MEDIAN_RT (msec)	4815.33	2055.63	0.73 (0.63–0.83)
2D_MANIPUL_MEDIAN_RT_for_CORRECT (msec)	5106.15	1938.67	0.76 (0.66–0.85)
VERBAL_REAS_MEDIAN_RT_for_CORRECT (msec)	4052.34	1121.4	0.77 (0.67–0.86)
LEARNING_CURVES_MEDIAN_RT (msec)	659.63	99.67	0.78 (0.69–0.87)
CHOICE_RT_MEAN_RT_for_CORRECT (msec)	593.43	89.34	0.89 (0.83–0.93)
SIMPLE_RT_MEAN (msec)	447.08	103.52	0.92 (0.88–0.95)

### Principal component analysis

Initial analyses of the objective and subjective sleep measures revealed that 113 out of 756 were significantly correlated, and therefore, we implemented a data reduction approach, as described by Balouch et al. (2022).

**FIGURE 3:**
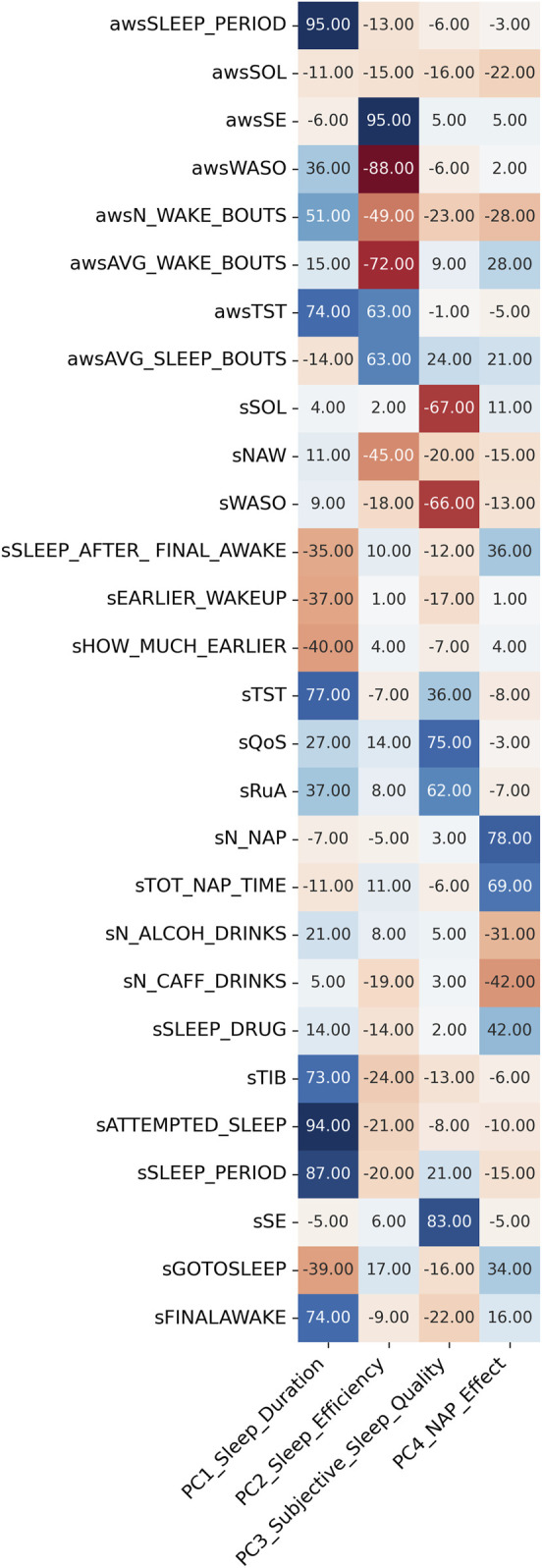
Principal component analysis of the sleep measures. Principal component analysis of the sleep measures identified four components. The strength of the contribution of the subjective and objective sleep measures to each component is indicated by color. Blue, positive weighting. Red, negative weighting.

Principal component analysis was performed, including 28 of the 31 objective (actigraphy) and subjective sleep diary variables (Table 1; Supplementary Figure S2). Three variables were excluded due to redundancy with other variables. The first four components were selected using the parallel analysis method, accounting for 54% of the total variance in the sleep variables, and were interpreted and labeled as follows: 1) sleep duration, 2) sleep efficiency, 3) subjective sleep quality, and 4) nap effect (Figure 3). The contribution of the variables ordered by sleep component is shown in Supplementary Figure S2. Intra-individual variation in sleep components is shown in Figure 2.

### Mixed-model analysis

**FIGURE 4:**
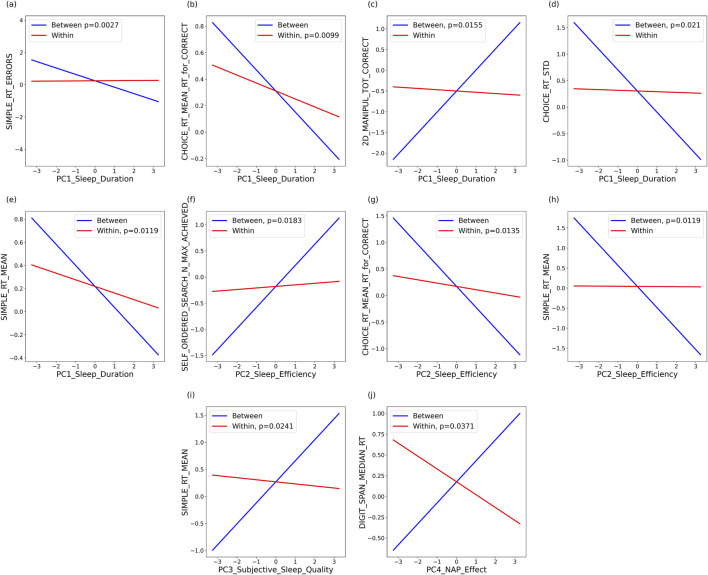
Modeled associations between daytime measures and principal components (PCs) of sleep for those associations for which the mixed model generated a significant effect of the principal component. Modeled associations were created for a female individual in her 70s. PC1: sleep duration and **(a)** simple reaction time errors; **(b)** choice reaction time, mean for correct trials; **(c)** 2D manipulation task (total correct trials); **(d)** standard deviation of choice reaction time; and **(e)** mean of simple reaction time. PC2: sleep efficiency and **(f)** self-ordered search task (maximum level achieved); **(g)** choice reaction time, mean for correct trials; and **(h)** mean of simple reaction time. PC3: subjective sleep quality and **(i)** mean of simple reaction time. PC4: NAP effect and **(j)** median of reaction time for the digit span task.

Mixed models were conducted with each cognitive measure as the dependent variable, along with age, sex, BMI, AHI, time, and sleep component as fixed effects. To assess the effects of both intra-individual and inter-individual variation in sleep on cognition for each sleep component, the individual’s average of that variable was used to model inter-individual effects, and the deviation from the mean was used to model intra-individual effects. There was a significant effect of time (study day) for several variables (60 out of 76 models; each model included a daytime cognitive variable and a principal component of sleep), which most likely reflects learning/practice effects, as illustrated in Figure 2 for total correct on the verbal reasoning task. Out of 19 cognitive variables, a significant effect of age (sleep components 1, 3, and 4) and AHI (sleep components 1, 3, and 4) was only observed for one variable (paired associate maximum score). Figure 4 provides examples of modeled associations for a female individual in her 70s for those associations for which the mixed model generated a significant effect of the principal component.

For the absolute (inter-individual) data, there was a significant effect of sleep duration (sleep component 1) on accuracy (simple RT errors, estimate = −0.397, F_(1,28)_ = 10.82, *p* = 0.0027; 2D manipulation total correct, estimate = 0.5076, F_(1,28)_ = 6.65, *p* = 0.0155) and reaction time (choice RT standard deviation, estimate = −0.3967, F_(1,28)_ = 5.98, *p* = 0.021). Increased sleep duration was associated with quicker response time and greater accuracy. There was a significant effect of sleep efficiency (sleep component 2) on reaction time (simple RT mean, estimate = −0.5247, F_(1,28)_ = 7.23, *p* = 0.0119) and maximum achieved (self-ordered search maximum score, estimate = 0.402, F_(1,28)_ = 6.28, *p* = 0.0183). Greater sleep efficiency was positively associated with faster reaction times and better performance.

For the deviation-from-the-mean (intra-individual) data, there was a significant effect of sleep duration (sleep component 1) on reaction time [choice RT mean (correct trials), estimate = −0.0601, F_(1,340)_ = 6.73, *p* = 0.0099; simple RT mean, estimate = −0.0572, F = 6.4, *p* = 0.0119], with longer durations associated with faster responses. Reaction time was also significantly affected by sleep efficiency (sleep component 2) [choice RT mean (correct trials), estimate = −0.0622, F_(1,340)_ = 6.16, *p* = 0.0135], subjective sleep quality (sleep component 3) (simple RT mean, estimate = −0.03816, F_(1,338)_ = 5.13, *p* = 0.0241), and naps (sleep component 4) (digit span RT median, estimate = −0.155, F_(1,343)_ = 4.38, *p* = 0.0371). Thus, greater sleep efficiency, better sleep quality, and increased naps were associated with quicker reaction times (see Supplementary Table S1).

## Discussion

Simultaneous assessment of sleep and cognition over 14 consecutive nights and days revealed that, in older people, both the inter-participant and intra-participant variations in sleep are associated with inter- and intra-participant variations in certain aspects of cognition. Sleep duration and sleep efficiency were the most prominent predictors of cognition. Longer sleep duration and higher sleep efficiency were associated with shorter reaction times for both inter- and intra-associations. Variations in many other aspects of cognition were not significantly associated with sleep in either the intra- or inter-analyses of association.

Our initial analyses focused on the intra- vs. inter-individual variation in sleep variables and cognitive variables. Subjective measures of wake in the time-in-bed period, such as sSOL, sSE, and sWASO, in general, had a lower ICC than objective measures of sleep continuity, for example, awsSE and awsWASO. This suggests that although objective sleep continuity, as measured using the Actiwatch, was relatively stable within an individual, the recall of their sleep episode was poor and varied from night to night. Our observed ICC values for objective sleep (0.35–0.65) and subjective sleep (0.11–0.73) are comparable to those previously observed in older cohorts (Balouch et al., 2022). PCA revealed that four principal components accounted for 54% of the total variance in the sleep variables. This is lower than that in the comparable study by Balouch et al. (2022), in which four components of sleep explained 75% of the variation in sleep variables. However, the identified components and their composition, i.e., the contribution of the various sleep features, were very similar in the two studies. Comparable analyses of the cognitive variables revealed that while reaction time was more consistent within an individual from day to day (higher ICC values), accuracy showed more daily variability (lower ICC values).

The relationship between sleep and cognitive function may be particularly relevant in the context of dementia as it could help predict its incidence, track its development, or target sleep as an intervention to slow disease development. Previous studies have observed associations between cognitive decline or incident dementia with sleep duration, sleep continuity, SWS, and REM sleep (Lucey et al., 2019; Sani et al., 2019; Winsky-Sommerer et al., 2019; Mander et al., 2022; Zhang et al., 2022; Li et al., 2023). In the present study, exploration of the relationship between sleep duration and cognitive performance revealed that, when comparing participants, increased sleep duration was associated with faster reaction times and greater accuracy. Within participants, higher sleep durations were associated only with faster reaction times. Our ICC analysis revealed that accuracy showed more day-to-day variations within individuals than reaction time; the lack of significant effects of sleep components on intra-participant accuracy suggests that factors other than sleep may be influencing this aspect of performance, such as stress and physical activity (Stawski et al., 2011; Phillips et al., 2016; Yang et al., 2025).

Our observation of positive effects of increased sleep duration on performance is consistent with two previous studies in older adults, demonstrating that poorer verbal fluency was significantly associated with lower self-reported sleep duration (Alves et al., 2021) and that actigraphic sleep duration ≥6 h was associated with improved episodic memory scores and faster reaction times (Bloomberg et al., 2024). However, several other studies in community-dwelling older adults (>60 years) have observed a negative relationship between long sleep durations and performance. Actigraphic sleep durations ≥8 h have been associated with poorer performance on a working memory task (single assessment) (Okuda et al., 2021), lower MMSE scores (indicative of higher cognitive impairment) and greater odds of impaired verbal fluency (Spira et al., 2017), and worse executive function (Winer et al., 2021). Long self-reported sleep durations were associated with lower MMSE scores (Kondo et al., 2021), along with impaired verbal memory, semantic fluency, working memory, and processing speed (Low et al., 2019). Long self-reported sleep duration was also associated with an increased risk of cognitive decline, whereas a late mid-sleep time appeared to be protective (Suh et al., 2018).

Previous studies have reported a U-shaped association between sleep duration and cognition, with both shorter and longer durations associated with poor performance (Guo et al., 2025). In this study, we also ran the model with a quadratic term to accommodate this feature; however, it was only significant in very few cases, and therefore, we did not present it here. A meta-analysis of 18 cross-sectional and prospective studies in older adults (>55 years) observed that both long and short sleep durations were associated with poor cognitive function, in particular working memory, verbal memory, and executive function (Lo et al., 2016). In a 7-day study in which older adults reported sleep duration and completed a daily numerical working memory task, inter-participant associations revealed that both shorter and longer sleep durations were associated with worse performance (Lucke et al., 2022). Within-participant associations revealed that, overall, night-to-night variations in sleep duration were not significantly associated with performance; however, for those participants who slept 1 hour less than the average participant, sleeping less than usual was associated with poorer performance (Lucke et al., 2022). In people living with Alzheimer’s disease (PLWA), both longer and shorter sleep durations were associated with poor performance in a task probing attention and calculation, but with fewer memory errors (Balouch et al., 2022). Long sleep duration has been shown to be a risk factor for cognitive decline (Gildner et al., 2019), and in older adults (65–85 years), self-reported short sleep duration (≤6 h) is associated with increased amyloid-β burden and impaired memory (Winer et al., 2021).

In addition to considering the duration of sleep per se, regularity of sleep is also an important factor for health and cognitive performance (Sletten et al., 2023). In a Biobank study (n = 60,977), higher sleep regularity (actigraphy) was associated with a 20%–40% lower risk of all-cause mortality and was a stronger predictor of mortality risks than sleep duration (Goodman et al., 2023). In this study, we observed that variable sleep duration was associated with increased reaction time, and variable subjectively reported wake time was associated with increased errors (see Supplementary Figure S3). A previous study in older adults (≥60 years) also revealed a significant correlation between the SD of sleep timing and number of errors (Okuda et al., 2021).

We also observed a relationship between sleep efficiency and cognitive measures, whereby lower sleep efficiencies were associated with slower reaction times and reduced accuracy, both within intra- and inter-individuals. Our observations are consistent with previous studies in older adults, in which increased actigraphic sleep disruption (e.g., higher WASO and number of awakenings) was associated with poorer episodic memory (Yeh et al., 2021), and higher sleep continuity was associated with reduced daytime sleepiness and fewer memory errors the subsequent day in PLWA (Balouch et al., 2022). Increased variability in actigraphic sleep efficiency was associated with worsening cognitive performance (Sakal et al., 2024). A recent meta-analysis that included 72 cross-sectional and within-participant articles explored the relationship between objective sleep (measured with PSG or actigraphy) and cognition in healthy older adults (Qin et al., 2023). The authors concluded that measures of sleep continuity, but not sleep duration, were significantly associated with cognition, while better performance (memory and executive function) was associated with lower WASO, higher sleep efficiency, and reduced restlessness. The authors suggest that the lack of relationship between sleep duration and cognitive parameters may be because the studies include total sleep time (TST) as a linear parameter rather than a quadratic or j-shaped curve, with durations <6 or >7 h associated with worsening cognition.

A recent study explored the relationship between night-to-night variations in sleep duration, efficiency, and quality with processing speed on the subsequent day over 3 weeks in younger and older adults (Schwarz et al., 2025). Intra-individual effects were observed for sleep duration (actigraphic) and sleep quality (subjective), with shorter-than-average durations and poorer sleep quality predicting worse performance the following day. No significant inter-individual effects were observed for the sleep variables and performance. Their observations of the intra-individual association of both sleep duration and quality with performance are consistent with our findings; however, their lack of association between sleep efficiency and cognition differs from the findings of our study.

## Limitations

The main limitation of the current study is that, due to the field-based nature of data collection for up to 14 days, sleep was assessed using actigraphy rather than the gold-standard approach of polysomnography. However, the ability to record data in a free-living environment is a beneficial aspect. Another limitation is the small sample size, which may indicate that the observed results are not generalizable to the wider population. In addition, we did not apply corrections for multiple testing as this was an exploratory study; nevertheless, our results are in accordance with those in the previously published literature.

## Future implications

Our demonstration that sleep and cognitive data can be collected from community-dwelling older adults over multiple days provides opportunities for future studies using these approaches. The ability to conduct long-term (days to weeks to months) assessments of sleep and daytime function in different clinical populations, including those living with dementia, will enable the tracking of disease progression/symptom presentation and explore mechanistic relationships between sleep and disease. Ultimately, this may allow for the development of sleep-targeted interventions to enhance daytime function and improve quality of life.

## Conclusion

We have demonstrated the feasibility and acceptability of conducting remote, longitudinal assessments of sleep and cognition in community-dwelling older adults. We have identified that measures of both sleep duration and sleep efficiency are associated with measures of performance, including speed and accuracy, and can explain both inter- and intra-individual variations. However, aspects of cognition related to learning, visual memory, verbal reasoning, and verbal fluency were not associated with sleep. The sensitivity of certain aspects of daytime function to spontaneous variations in sleep implies that, for vulnerable populations, interventions targeting sleep aspects such as sleep continuity may lead to improvement in daytime function.

## Data Availability

The raw data supporting the conclusions of this article will be made available by the authors, without undue reservation.

## Supplementary material

SUPPLEMENTARY FIGURE S1 Boxplots of inter and intra-individual variation for objective sleep efficiency (awsSE), subjective sleep efficiency (sSE), and total correct answers in the verbal reasoning task (VERBAL_REAS_TOT_CORRECT) in 35 participants. In the left-hand panel, the raw data for each participant are plotted sorted by objective sleep efficiency (awsSE %). The horizontal lines represent the median, the filled circles are individual points, the boxes represent the central 50% of the data, and the vertical lines indicate the maximum/minimum values excluding outliers. In the right-hand panels the data are plotted separately for each of the 14 days. Variables were first expressed as deviation from the average in each participant and then averaged across participant for each day. Please note that performance in the verbal reasoning task improved over the course of the 14 days, whereas the sleep variables remained constant.

SUPPLEMENTARY FIGURE S2 Principal component analysis of the sleep measures. Principal components analysis of the sleep measures identified four components. The strength of the contribution of the subjective and objective sleep measures to each component is indicated by colour. Blue: positive weighting. Red: negative weighting. The variables are ordered by the strength of their contribution (absolute value) to each component.

SUPPLEMENTARY FIGURE S3 Heatmap of Pearson correlation between mean cognitive performance variables and standard deviation of objective and subjective measures of sleep regularity.
