# Supplementary figures and images for: Within- and between-individual associations between sleep and cognition in older community-dwelling individuals
Source: Front Aging. 2026 Jan 22;6:1650312. doi: 10.3389/fragi.2025.1650312 (PMC12873480; doi:10.3389/fragi.2025.1650312)

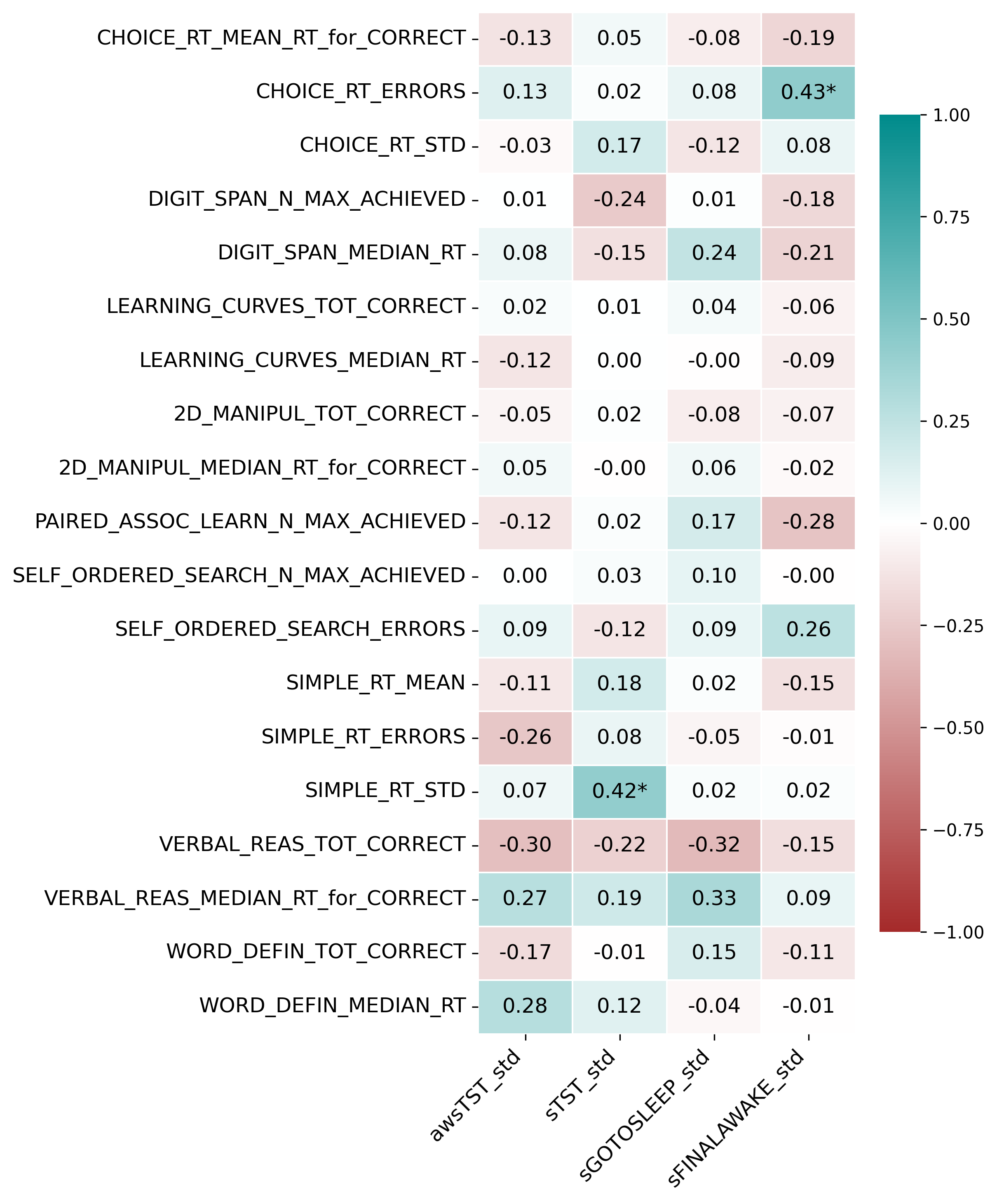

Supplement: Supplementary file 1 [file Image3.tiff]

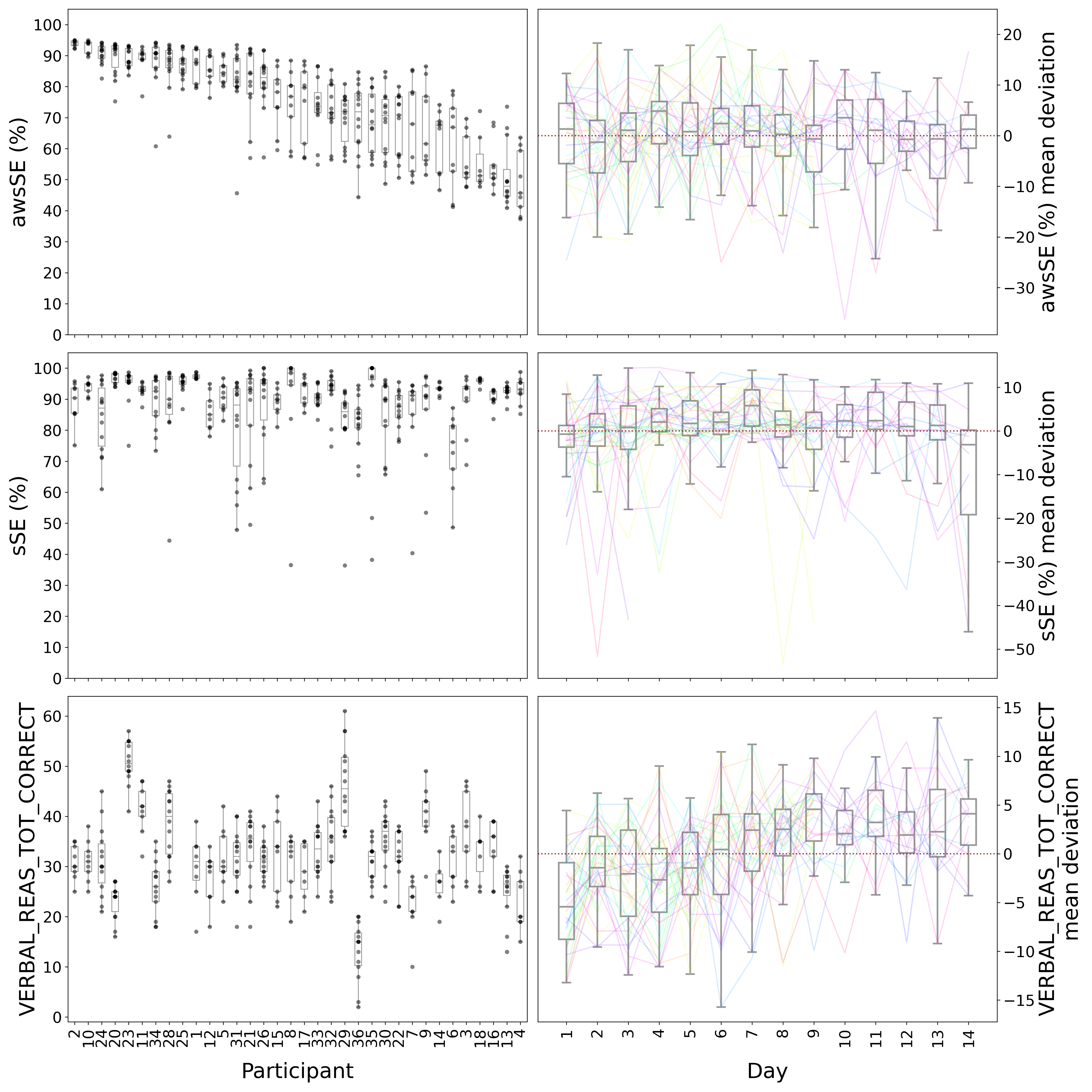

Supplement: Supplementary file 2 [file Image1.tiff]

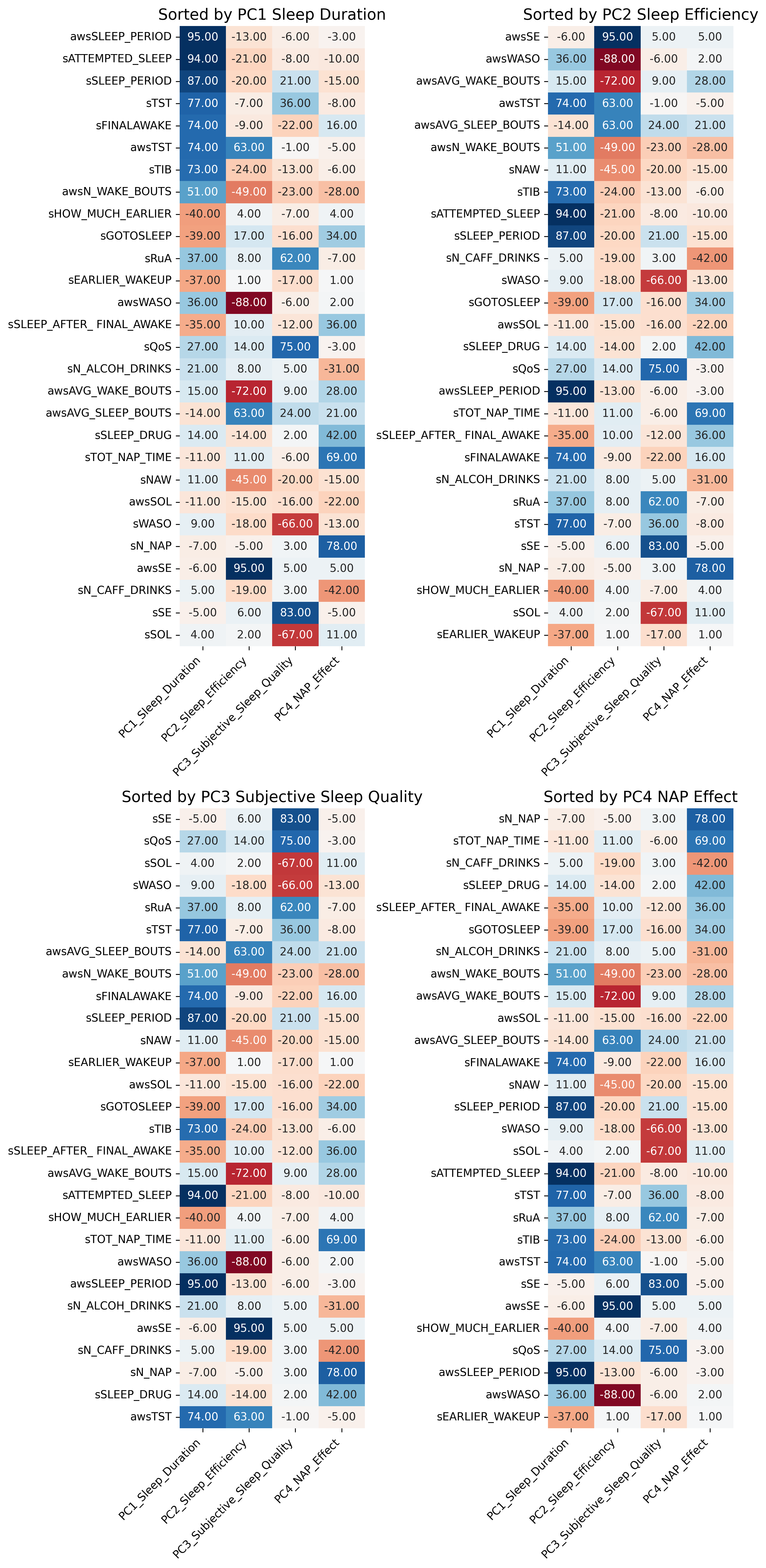

Supplement: Supplementary file 4 [file Image2.tiff]
